# Knee Abduction Angles and Landing Kinematics in Badminton Jump Smash: A Study of ACL Injury Risk Factors

**DOI:** 10.3390/bioengineering12040343

**Published:** 2025-03-26

**Authors:** Ming Wei Yeap, Yuvaraj Ramasamy, Juliana Usman, Mark King, Rizal Razman

**Affiliations:** 1 Division of Research and Innovation, National Sports Institute of Malaysia, Kuala Lumpur 57000, Malaysia; mingweiyeap27@gmail.com; 2 Faculty of Engineering, Universiti Malaya, Kuala Lumpur 50603, Malaysia; juliana_78@um.edu.my; 3 Division of Sports Performance, National Sports Institute of Malaysia, Kuala Lumpur 57000, Malaysia; yuvarajramasamy78@gmail.com; 4 Centre for Applied Biomechanics, Faculty of Engineering, Universiti Malaya, Kuala Lumpur 50603, Malaysia; 5 School of Sport, Exercise and Health Sciences, Loughborough University, Loughborough LE11 3TU, UK; m.a.king@lboro.ac.uk; 6 Faculty of Sports and Exercise Science, Universiti Malaya, Kuala Lumpur 50603, Malaysia

**Keywords:** kinematics, knee, junior, forehand jumping smash, single leg landing

## Abstract

Anterior cruciate ligament (ACL) injuries can occur in non-contact conditions (e.g., jump-landing) and are common among junior badminton players. The knee abduction angle has been widely identified as a biomechanical risk factor that likely contributes to this injury mechanism. Purpose: This study aims to examine the relationship between the trunk and lower limb landing kinematics and the peak knee abduction angle following a jumping smash. Method: Twenty-one male junior badminton players performed jump smashes on an instrumented badminton court. Anthropometry was measured; trunk and lower limb single-leg landing kinematics and kinetics were collected using a motion capture system. Pearson’s correlation was performed to identify the variables significantly correlated to peak knee abduction angle, followed by stepwise multiple regression to identify the most important combination of predictors. Results: Regression analysis showed that knee external rotation angle at foot contact and peak knee internal rotation angle were associated with peak knee abduction angle. A separate analysis also showed that landing time was positively associated with peak knee abduction angle. Conclusions: Assessing ACL injury risk and developing injury prevention strategies for jump landings in badminton should focus on knee motion in the frontal and transverse planes, as well as landing time.

## 1. Introduction

Badminton is a fast-paced sport that demands short bursts of movement involving frequent quick starts and stops, lunges, jumps, and abrupt sharp changes in direction [1]. These rapid acceleration and deceleration movements potentially stress the lower extremities, increasing the risk of injury [1]. In recent years, there has been growing attention on anterior cruciate ligament (ACL) injuries in badminton, particularly after more ACL ruptures were reported among top-level players [2]. A retrospective analysis of badminton players found that 37% of reported injuries involved the ACL [3]. These injuries predominantly occur through two major mechanisms: single-leg landing after an overhead stroke and plant-and-cut maneuvers. Another study involving 539 badminton players found that competitive players sustain most ACL injuries in the rear court [4], supported by a recent study on world elite players, which revealed that 75% of reported ACL injury movements were associated with jumps [5]. Younger players are particularly affected, showing an overrepresentation of injuries in the rear court [3,4]. This is likely due to their forceful playing style, characterized by higher and more frequent jumps that generate greater forces around the knee joint. The smash, a key component of a player’s repertoire constituting 54% of the winning shots in international competition [6], exemplifies this risk. While there is no consensus in the literature on whether a pre-jump enhances smash speed, a higher contact point with the shuttlecock enables a steeper smash angle. However, the landing poses a common risk of injury [1].

ACL injury is a debilitating condition among athletes. Previous research has shown that ACL injuries involve multiplanar biomechanical movements at the knee joint, with peak ACL load occurring approximately 30–50 milliseconds after initial foot contact [7]. In line with this, kinematic analysis of 10 non-contact ACL injury cases using three-dimensional video showed consistent hip, knee, and ankle movements [8,9]. An immediate knee abduction of 12° was observed within 40 milliseconds of initial foot contact, accompanied by internal knee rotation [8]. The authors suggest that valgus loading and internal rotation are combined movements and key factors in the ACL injury mechanism [8,10]. Additionally, all 10 injured athletes displayed a low knee flexion angle (~23°) [8], supporting the idea that reduced knee flexion (<30°) compromises the mechanical advantage of the hamstrings relative to the quadriceps, potentially increasing anterior tibial shear forces [11]. Furthermore, the hip joint in all injured athletes remained in a flexed and internally rotated position during the first 40 milliseconds after initial contact [9]. These athletes consistently landed with a heel strike, showed minimal dorsiflexion, and transitioned to a flat-foot position within 20 milliseconds [9]. Similar ankle kinematics were also observed in 73% of male professional football players who sustained ACL injuries [12]. Recurring sprains of the ankle can lead to chronic ankle instability, and this is not uncommon among athletes [13]. Dysfunction or instability in the ankle can lead to compensatory movements in adjacent joints due to the distal to proximal influence along the kinetic chain. Increased hip and knee extension moments, decreased hip flexion angles, as well as increased trunk lateral flexion angles were observed in individuals with chronic ankle instability during landing tasks [13]. These biomechanical alterations are linked to an increased risk of ACL injuries. In fact, previous studies have demonstrated that the knee abduction angle (KAA) is a potential predictor highly comparable to the knee abduction moment (KAM) [7,14]. Because KAA can be more easily observed clinically than KAM, it provides a simpler means to assess the effectiveness of injury risk reduction through intervention training [7].

In badminton, which emphasizes single-arm dominance, players often adopt an asymmetric posture to maintain balance, potentially increasing the likelihood of landing on one leg from a jump and landing in a dynamic valgus position [15]. Most players (~90%) injured the knee opposite their racket-hand side [3], consistent with the evidence that players typically land on the contralateral limb to the racket-hand side [15,16]. Landing on a single leg evidently exposes the landing knee to a more precarious situation, as this stance provides a smaller base of support. Moreover, the absorption of all impact forces relies solely on the muscle actions of one leg, potentially straining the ACL while decelerating joint movements [17]. A smaller hip and knee flexion angle, higher peak knee flexion angular velocity, and a smaller ankle dorsiflexion angle were also observed when landing on one leg compared to landing with both legs [17].

Meanwhile, during a forehand jump smash, the forward rotation of the trunk and the reaching movement of the arm toward the shuttlecock often lead to lateral trunk tilt upon landing. This shifts the center of mass over the stance limb during landing, potentially causing greater abduction and impact loading in the knee [18]. Several past studies investigating trunk movement and landing biomechanics have found a negative correlation (*r* = −0.30, *p* < 0.001) between trunk flexion angle and knee abduction [19], with a reduced trunk lateral flexion angle significantly reducing the incurred knee abduction (*p* < 0.01) [20]. Concurrently, a significant association was found between a smaller ankle plantarflexion angle at foot contact and an increased KAM during landing [21]. Previous studies comparing injured and non-injured data identified a smaller knee flexion angle and angular velocity, greater trunk lateral flexion angle, and increased ground reaction force (GRF) as significant contributing risk factors [22,23].

A previous study on badminton highlighted that increased knee valgus during single-leg landings, particularly after executing an overhead stroke, may predispose athletes to ACL injuries [24]. In another study, Hung et al. [15] explored the effectiveness of different landing strategies during badminton footwork training, specifically focusing on the backhand side lateral jump smash. Research showed that landing on a single leg after a backhand lateral jump smash increased the risk of lateral ankle sprains, particularly when fatigued [25]. This was evidenced by a higher landing inversion angle, peak inversion, and internal rotation moment compared to backhand rear-court jump smash. However, research on this topic remains limited, and more studies are needed to fully understand the injury mechanisms and provide insights for effective injury prevention strategies. In contrast, much more attention has been given to investigating how smash kinematics and shuttlecock-racket impact location contribute to smash performance [6,26,27]. Additionally, many studies on jump landing biomechanics utilized general tasks, such as the drop jump, countermovement jump, and stop jump, to examine landing mechanics and the associated ACL impact loading and/or to better understand lower limb biomechanics during specific maneuvers [23,28]. However, these tasks do not always replicate sport-specific movements.

It is important that the jump-landing tasks used in studies closely mimic the specific movements of interest to achieve the highest possible ecological validity. Indeed, determining trunk and lower limb kinematic risk factors associated with peak KAA during a forehand jump smash is crucial. We anticipate that the findings will provide valuable insights for coaches, athletes, and practitioners to explore a range of preventive measures aimed at reducing the incidence of ACL injuries. Therefore, the purpose of this study was to examine how trunk and lower limb landing kinematics, along with spatiotemporal and anthropometry measures, relate to peak KAA during a forehand jump smash. We hypothesized that players with reduced knee flexion and increased knee internal rotation and trunk lateral flexion angles would exhibit a greater KAA during landing. Secondly, a taller physique and longer tibia length would be associated with a greater KAA during single-leg landing following a jumping smash.

## 2. Materials and Methods

### 2.1. Participants

The sample size was estimated using G-power software 3.1.9.4 (University of Düsseldorf, Düsseldorf, Germany), and the overall sample size needed was 19 to detect correlation with an alpha of 0.05 and 80% statistical power [29]. Twenty-one male junior badminton players (mean ± SD: age, 14.8 ± 1.5 years; standing height, 167.9 ± 7.4; body mass, 57.3 ± 8.9 kg; BMI, 20.2 ± 2.3) who trained full time at a professional badminton club or national sports school were recruited. None had a history of hip, knee, or ankle surgery or injury 6 months prior to the testing. All participants were free from any diseases, injuries, or symptoms that could interfere with their performance at the time of data collection. While this criterion was used to minimize the influence of recent injuries, it is important to note that it does not account for chronic or pre-existing conditions that may have been present. The study was approved by the Human Research Ethics Committee of the National Sports Institute of Malaysia (ISN), with written informed consent obtained from participants and their parents/guardians.

### 2.2. Experimental Procedures

The motion capture system was calibrated at least an hour before the data collection started. In the beginning, body anthropometry was measured. After 10 min of self-administered stretching, markers were attached to the bony landmarks of the participant. Each participant was then given 20 min for warmup and court familiarization before data collection.

**Figure 1:**
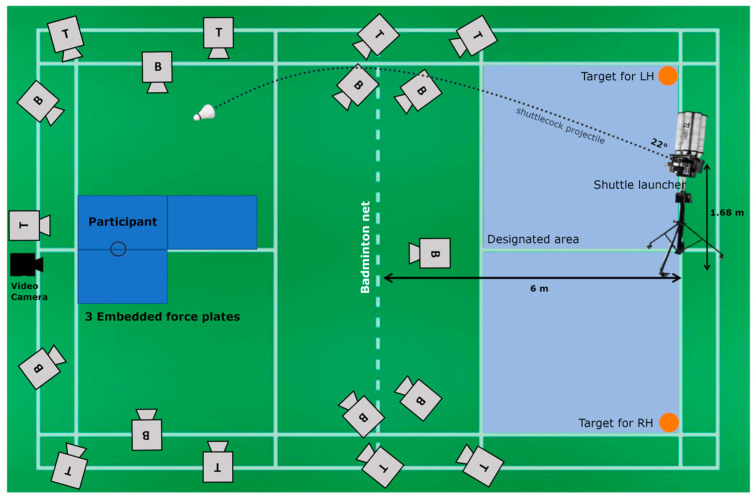
Laboratory setup for the jump smash test. B: bottom, T: top, LH: left-handed, RH: right-handed.

A Knight Trainer (Black Knight, Montreal, QC, Canada) shuttle launcher was used to replicate a high serve/lift to the participants [26]. The shuttlecock launcher was positioned at a height of 1.68 m from the ground to the serving wheel and angled at 22° from horizontal (see Figure 1). The shuttlecock launching protocol was set at a speed of 140 km/h, and the frequency was set at 10 per 30 s. The position and setup of the shuttlecock launcher were standardized and not adjusted based on players’ height. The mean height of the shuttlecock trajectory was 2.81 ± 0.14 m between the player’s peak center of mass height and shuttlecock contact. The participants performed 2 sets of smashes (30 s per set) with 2 min rest in between sets. Each participant performed 20 jump smash trials in total on the same side as their handedness. The five fastest (indicated by shuttlecock speed) successful trials with single-leg landing were chosen for each participant for further analysis; these were identified by the following criteria: (a) the shot crossed the net and hit the designated area on the opposite court, (b) the participant took off and landed within the force plates embedded and positioned level to the court floor, and (c) the jump height of the smash trials was at least 30 cm vertically (identified by the maximum vertical displacement of the L_IPS marker from that of static trial standing position).

### 2.3. Instrumentation and Data Collection

The smash test was conducted under controlled conditions on an instrumented standard badminton court at the Sports Biomechanics Laboratory in ISN (see Figure 1). Kinematics data were recorded at 400 Hz using 18 Oqus 7+ series infrared cameras (Qualisys AB 411 05, Gothenburg, Sweden) and one high-speed video camera; GRF data were recorded at 300 Hz using three Kistler force plates (9287CA) (900 mm × 6000 mm) positioned leveled with the floor.

**Figure 2:**
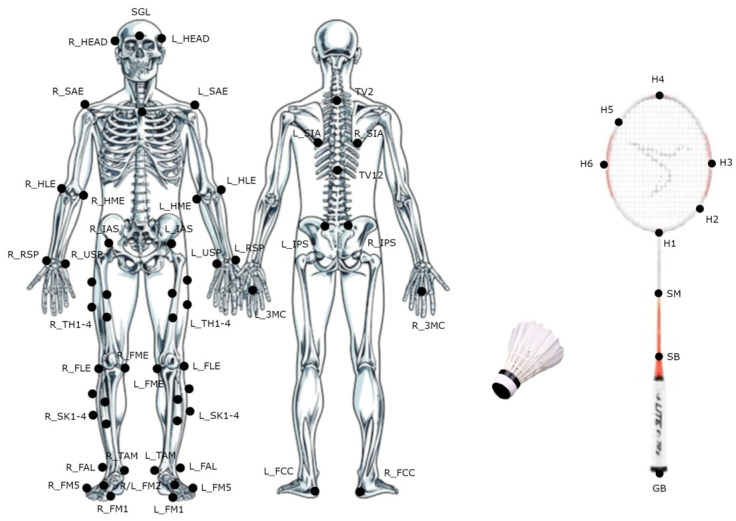
Markers placement on bony landmarks, badminton racket, and shuttlecock.

Fifty-four 12.5 mm reflective markers were attached to key bony landmarks using Qualisys double-sided and Hypafix non-woven adhesive tape to create a 15-segment representation in Visual 3D v6.00.18 (C-Motion Inc., Germantown, MD, USA) [26]. Each racket was equipped with one 12.5 mm marker on the bottom of the handle and eight pieces of retro-reflective tape (10 mm^2^) on the racket head and shaft (Figure 2). All participants used their own rackets for the test. New Yonex Aerosense 30 shuttlecocks were used for data collection, with a circle-reflective tape (diameter 19 mm) attached to the tip of the shuttlecock. Mis-shaped or broken shuttlecocks were discarded.

Anthropometric measurement was performed in accordance with the International Society for the Advancement of Kinanthropometry (ISAK) guidelines. The anthropometric data included body mass, body height, skinfold, girth, and limb length.

### 2.4. Data Analysis and Reduction

Foot contact (FC) was determined by the instant when the vertical GRF exceeded 20 N, while the end of the landing phase was defined as the instant of maximum knee flexion. Landings were categorized as “single leg” if the delay in the arrival of the second foot to the ground after the first foot contact was greater than 0.033 s [30]. Only the first foot contact was analyzed for each trial. Joint angles were calculated as Cardan angles, where an xyz rotation sequence was used for all angles except for the thorax angle, for which a yxz rotation sequence was used [31].

Position data were labeled using Qualisys Track Manager (QTM2022.1: build 4260, Qualisys, Gothenburg, Sweden), and all body marker position data and force plate data were filtered in Visual 3D using a 4th-order, zero-phase, low-pass Butterworth filter with a cut-off frequency determined through a residual analysis in MATLAB R2022a (MathWorks Inc., Natick, MA, USA). A 15-segment model in Visual 3D was used to calculate descriptive kinematic variables. Processed data were time-normalized to 101 data points. Peak GRF was normalized to body weight. The variables selected were based on scientific evidence related to ACL injury risk factors and ACL injury outcomes [9,10,14,15,18,21,29,32]. The variables were categorized into anthropometry, spatiotemporal, and kinematics (see Section 3).

### 2.5. Statistical Analysis

All values are presented as means ± SD. The intraclass correlation coefficient (ICC) was calculated and interpreted with the following classifications adopted: poor (<0.4), fair (0.4–0.6), good (0.6–0.74), and excellent (>0.74) [33]. If high similarity within each participant was found (indicated by a good to excellent ICC value), the means of the five trials for each player were combined into a single time-history for further analysis. Data were tested for a normal distribution using the Shapiro–Wilk test, and the assumption of normality was met when *p* > 0.05.

A two-tailed Pearson product moment correlation test was used to detect significant associations between (a) anthropometry variables, (b) spatiotemporal variables, (c) kinematics variables, and KAA, respectively. Next, stepwise multiple regression analysis was performed to identify the significant predictors that contributed to the peak knee abduction angle during single-leg jump-smash landing. All statistical tests with variable significance were set at *p* < 0.05 and were performed using the statistical software IBM SPSS Statistics (v27, IBM Corp., Armonk, NY, USA).

## 3. Results

The descriptive data for kinematics, spatiotemporal, and anthropometry measures are presented in Table 1. All variables tested with interclass correlation were classified as good (0.6–0.74) or excellent (>0.74). Hence, the mean of the five trials of each player was combined into a single time-history for each variable for further analysis.

**Figure 3:**
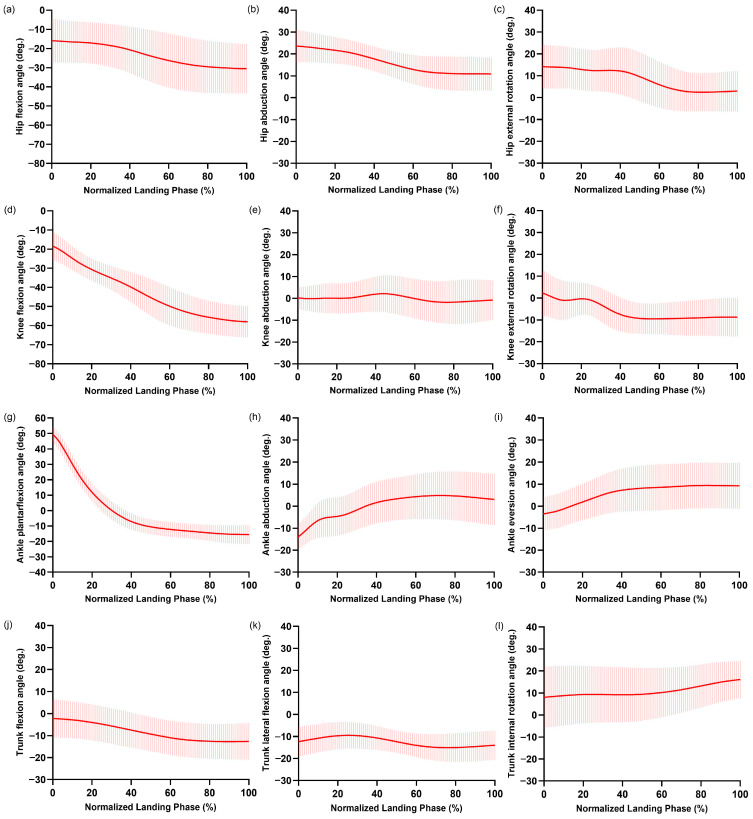
Average curves of the hip flexion (**a**); hip abduction (**b**); hip external rotation (**c**); knee flexion (**d**); knee abduction (**e**); knee external rotation (**f**); ankle plantarflexion (**g**); ankle abduction (**h**); ankle eversion (**i**); trunk flexion (**j**); trunk lateral flexion (**k**); trunk internal rotation (**l**) angles. Error bars indicate ± one standard deviation.

The average knee flexion angle increased from −18.4° at FC to −58.0° at the peak (Figure 3d). The hip flexion, hip abduction, hip external rotation, knee external rotation, ankle plantarflexion, and trunk flexion angles showed decreasing angles (Figure 3a–c,f,g,j), while the ankle abduction, ankle eversion, and trunk internal rotation angles showed increasing values (Figure 3h,i,l) from FC to the peak knee flexion. The trunk laterally flexed toward the contralateral side of the racket hand at a peak value of −16.8° (Figure 3k).

The results of Pearson correlation test and regression analyses are presented in Table 2 and Table 3, respectively.

### 3.1. Kinematics

Significant correlations were only found between the KAA and certain kinematics variables (pooled values: *p* < 0.018). In essence, only the knee external rotation angle at foot contact was found to be highly correlated with the KAA (*r* = −0.706), while the knee flexion angle at foot contact (*r* = −0.531), trunk lateral flexion angle at foot contact (*r* = 0.511), and peak knee internal rotation angle (*r* = 0.563) were moderately correlated with the KAA. When multiple regression was calculated for the KAA in the validation subsample of the participants, the predictors explained 56% of the variance in the criterion. The significant partial regressors were knee external rotation angle at foot contact (62%, *p* = 0.002) and peak knee internal rotation angle (38%, *p* = 0.041).

### 3.2. Spatiotemporal Measures

Jump height (*r* = −0.469, *p* = 0.032) and landing time (*r* = 0.475, *p* = 0.029) showed significant moderate correlations with KAA. Meanwhile, only landing time (*p* = 0.029) was included in the regression model, which explained 19% of the variance in the criterion.

### 3.3. Anthropometry

No significant association was found between KAA and any of the anthropometric variables (pooled values: *p* > 0.125).

## 4. Discussion

To the best of the authors’ knowledge, this is the first study to consider the kinematic, spatiotemporal, and anthropometric predictors of peak knee abduction angle in sub-elite junior male badminton players. Multiple regression analysis found that the significant predictors of peak KAA included knee external rotation angle at foot contact, peak knee internal rotation angle, and landing time. Meanwhile, Pearson’s correlation analysis revealed a weak relationship between peak KAA and jump height, and moderate relationships were found between peak KAA and knee flexion angle and trunk lateral flexion angle at foot contact. None of the anthropometry measures predicted peak KAA in this study.

During a forehand jumping smash in this study, players typically shift their body weight from back to front and land on the leg contralateral to the racket hand, which is consistent with the literature [3]. The single-arm dominancy nature of badminton with the arm position constrained by the racket influences lower limb dynamics and could likely increase the tendency of players to land on a single leg and in a dynamic valgus position [3,15].

The current findings showed that increased knee internal rotations (reduced external rotation) at foot contact and peak segment angle significantly contributed to a higher KAA. This is consistent with evidence from the literature that explains the majority of ACL injury mechanisms in females [14], suggesting that male junior badminton players also demonstrated a certain extent of knee internal rotation when in a valgus position. The knee rotating internally soon after foot contact could possibly be due to the “screw-home movement” when the knee is minimally flexed [34]. It was suggested that excessive internal tibial rotation during landing could be due to lower contraction activity of the external rotator muscle of the knee, which is insufficient to restrict the internal rotation [35]. A cadaveric study demonstrated that the maximum load and peak strain on the ACL (~11.5%) occurred when combined rotational forces were applied to the knee joint, with particular focus given to the coupling between tibial internal rotation and KAM [36].

Players should land with a neutral knee position with minimum rotation. According to the literature, the knee also could rotate externally during dynamic knee valgus testing [34] and at the time of ACL injury [32]. It was suggested that the combination of knee abduction and external rotation also could lead to ACL injury due to the impingement at the ACL on the femoral condyle [32]. A recent study proposed that impingement occurs at the midportion of the ACL when the knee is externally rotated past 15° and flexed between 30 to 40° [37]. Although, in general, males had greater joint stiffness and lower rotational joint laxity than females, it is not easy to control muscle activity voluntarily during landing from a maximal jump smash in the sense of maintaining a neutral position of the knee (reducing abduction and internal rotation), especially when fatigued [35]. Forceful internal rotation of the knee could be hard to resist even when the knee muscles have been maximally activated [38]. Therefore, it is crucial for players to develop good strength for the knee rotators and knee adductors muscles, excellent neuromuscular control, as well as proprioception, which could help reduce the rate of non-contact ACL injuries.

Additionally, the current results showed that greater landing time is associated with greater peak KAA could be due to the lengthened period that allows the lower limbs to move through a greater range of motion, including knee abduction. In contrast, Hewett et al. [14] found that the athletes who developed an ACL injury demonstrated a 16% lower landing time compared to the non-injured athletes. In general, landing with high ground reaction force over a short time (high impulse) imposes greater mechanical strain on the joints, which could lead to injury [32]. However, there is no direct evidence that a shorter landing time is associated with ACL injury. We suggest that players work on minimizing the landing impulse by lengthening landing time or reducing ground reaction force over a shorter period, without compromising their ability to quickly recover to the base position or the intended direction toward the shuttlecock.

The knee flexion and trunk lateral flexion angle at foot contact respectively showed moderate inverse and positive correlations with peak KAA during single-leg landings from the jump smash, which is consistent with the literature [19,39]. Stiff landing with a small knee flexion angle (<30°) has been widely shown to be associated with greater KAA and high ACL injury risk [11]. A previous study reported greater knee flexion in male badminton players than in their female counterparts during single-leg landing, which could be due to greater neuromuscular control and strength in males [14]. On the other hand, in badminton, the shift of the trunk mass toward the contralateral side would elevate the height of the dominant shoulder, allowing the player to contact the shuttlecock at a higher point [40]. However, when the foot lands and fixes on the ground, the lateral trunk tilt could result in an external hip abduction moment on the contralateral limb, which then needs to be counterbalanced by an internal hip adduction moment. Such compensation may cause the knee to move medially and increase knee abduction motion during landing [14]. Consistent with the literature, our study demonstrated that the trunk lateral flexion angle at foot contact significantly predicts the peak knee abduction during single-leg landing. Overall, the lack of significant deviations in trunk flexion and lateral flexion angles throughout the cycle may be attributed to several factors. First, athletes may control and limit trunk movements to avoid losing balance and quickly prepare for directional changes [14,41]. Second, since the player’s start position and shuttlecock trajectory were predictable in this study, there was likely minimal lateral movement around the court during the jump smash, potentially limiting the range of trunk movement.

Interestingly, the anthropometry measures in this study were not able to predict peak landing KAA in the junior male players following a jump smash. This could be due to the participants recruited in this study being juniors and having different physical maturity levels [42]. They are still in their adolescent years, in which a growth spurt could occur at any time, and each individual has a different growth rate. Differences in the maturity of adolescents and youths could significantly influence anthropometric-related results [43]. Furthermore, the sample size in this study is relatively small compared to typical morphological-type studies that require a large number of participants for better normative data. Ideally, a taller and leaner physique with less adiposity gives badminton players physical and physiological advantages in their sports performance [44]. However, considering that longer limb lengths and heavier segments can generate greater torque at the joints, it is crucial to develop corresponding neuromuscular control and strength to meet the movement demands of the sport.

There are several limitations in this study. First, female players were not included due to practical constraints during data collection; therefore, a replicate study with female participants is warranted. We also did not consider smash under pressure or asking players to move quickly back to the base after landing. Moreover, we did not consider other biomechanical factors, such as muscle strength and muscle activation, among others, that might be confounding factors.

## 5. Conclusions

In conclusion, our results confirm the association of increased knee internal rotation (reduced knee external rotation) at foot contact and peak segment angle with a greater KAA during single-leg landing in junior male badminton players following a jump smash. Additionally, reduced knee flexion and increased trunk lateral flexion angles at foot contact were associated with greater KAA during landing, supporting the first hypothesis. The second hypothesis was rejected, as none of the anthropometry variables were associated with a greater KAA during a single-leg landing following a jump smash. We suggest that correct landing with a neutral knee position should be included in court training and physical conditioning sessions to strengthen the muscles and knee complex, which could potentially help reduce the risk of ACL injury when landing from a jump. During training, the coach could give verbal instructions (with reference to knee rotation based on toe direction) to the players for landing technique correction. Concurrently, video playback could also give ideas to the players about their natural landing technique. Our results also indicated that increased landing time was moderately correlated with a greater knee abduction angle in this study. A longer landing time may allow for increased joint motion (KAA); however, it is not a direct indicator of ACL injury. In general, a soft landing lengthens landing time and enables more knee flexion. However, because quick footwork backward and/or in a diagonal direction is often involved after the jumping smash during a game, we suggest future studies consider the footwork involved when investigating the landing mechanism following a jump smash. Careful consideration is required when designing injury prevention measures to avoid compromising performance. Moreover, future studies might also consider additional factors (e.g., muscle strength, coordination) to obtain more comprehensive insights into jump smash-landing mechanisms.

## Figures and Tables

**Table 1:** Descriptive data of the variables.

Categories	Variables	Mean ± SD
kinematics	ankle dorsi/plantarflexion angle at FC (°)	49.3 ± 5.3
	knee flex/extension angle at FC (°)	−18.4 ± 7.5
	knee int/external rotation angle at FC (°)	2.3 ± 10.7
	knee add/abduction angle at FC (°)	0.3 ± 5.1
	hip add/abduction angle at FC (°)	23.7 ± 7.4
	trunk lateral flexion angle at FC (°) ^a^	−12.3 ± 6.7
	peak ankle dorsiflexion angle (°)	−16.4 ± 5.7
	peak knee flexion angle (°)	−58.0 ± 8.2
	peak knee abduction angle (°)	4.7 ± 7.0
	knee abduction range of motion (°) ^b^	9.0 ± 3.2
	peak knee internal rotation angle (°)	−12.1 ± 7.1
	peak hip flexion angle (°)	−2.1 ± 8.6
	peak hip abduction angle (°)	23.7 ± 7.4
	peak hip external rotation angle (°)	16.6 ± 9.1
	peak trunk flexion angle (°)	−14.2 ± 8.2
	peak trunk lateral flexion angle (°) ^a^	−16.8 ± 6.4
	peak knee flexion angular velocity (°/s)	584.3 ± 127.8
	peak hip flexion angular velocity (°/s)	314.9 ± 93.0
spatiotemporal	maximum height of center of mass (cm)	139.75 ± 11.09
	jump height (cm)	54.57 ± 10.66
	jump height to standing height ratio	0.83 ± 0.04
	landing time (s)	0.14 ± 0.02
	time to peak GRF (s)	0.06 ± 0.01
	time to peak KAA (s)	0.08 ± 0.03
anthropometry	BMI	20.2 ± 2.3
	sitting height (cm)	88.2 ± 5.1
	waist circumference (cm)	69.9 ± 6.1
	glute circumference (cm)	87.6 ± 5.8
	thigh circumference (cm)	48.7 ± 3.7
	calf circumference (cm)	34.8 ± 2.2
	relative trunk length (cm)	52.5 ± 1.4
	arm length (cm)	77.0 ± 3.3
	leg length (cm)	100.6 ± 5.2
	tibial length (cm)	37.8 ± 2.1
	arm-to-leg ratio	0.77 ± 0.03
	bone density (g/mL)	1.08 ± 0.01
	body fat%	9.84 ± 3.83

Notes: ^a^ denotes lateral flexion with reference to the racket-hand side. ^b^ measured as the difference between the minimum and maximum angles observed during the landing phase.

**Table 2:** Pearson’s correlation between peak KAA and the selected variables.

Categories	Variables	*r*	*p*
kinematics	ankle dorsi/plantar flexion angle at FC (°)	−0.097	0.675
	knee flex/extension angle at FC (°)	0.531	0.013 *
	knee int/external rotation angle at FC (°)	−0.706	0.000 **
	hip add/abduction angle at FC (°)	0.099	0.671
	trunk lateral flexion at FC (°)	0.511	0.018 *
	peak ankle dorsiflexion angle (°)	0.417	0.060
	peak knee flexion angle (°)	−0.275	0.228
	peak knee internal rotation angle (°)	0.563	0.008 **
	peak hip flexion angle (°)	−0.027	0.446
	peak hip abduction angle (°)	0.154	0.506
	peak hip external rotation angle (°)	−0.077	0.739
	peak trunk flexion angle (°)	0.404	0.069
	peak trunk lateral flexion angle (°)	0.283	0.214
	peak knee flexion angular velocity (°/s)	−0.014	0.953
	peak hip flexion angular velocity (°/s)	0.235	0.305
spatiotemporal	maximum height of center of mass (cm)	−0.350	0.120
	jump height (cm)	−0.469	0.032 *
	jump height to standing height ratio	−0.432	0.051
	landing time (s)	0.475	0.029 *
	time to peak GRF (s)	0.301	0.185
	time to peak KAA (s)	0.332	0.141
anthropometry	body mass index (BMI)	0.081	0.728
	sitting height (cm)	−0.163	0.480
	waist circumference (cm)	0.152	0.510
	glute circumference (cm)	0.050	0.831
	thigh circumference (cm)	−0.194	0.400
	calf circumference (cm)	0.081	0.726
	relative trunk length (cm)	−0.149	0.519
	arm length (cm)	0.265	0.246
	tibial length (cm)	0.118	0.610
	leg length (cm)	−0.070	0.762
	arm-to-leg ratio	−0.090	0.697
	bone density (g/mL)	−0.346	0.125
	body fat percentage (%)	0.346	0.126

Notes: * denotes *p* < 0.05; ** denotes *p* < 0.01.

**Table 3:** Stepwise regression model summary.

Categories	Predictors Model	R	R^2^	Adj. R^2^	F	Beta	*p*
kinematics	* knee int/external rotation angle at FC	0.777	0.604	0.560	13.752	−1.662	0.002
	* peak knee internal rotation angle					1.010	0.041
	knee flexion angle at FC					0.085	0.729
	trunk lateral flexion angle at FC					0.072	0.718
spatiotemporal	* landing time	0.475	0.226	0.185	5.547	0.475	0.029
	jump height					−0.377	0.068

Notes: * denotes variables entered the regression model. FC: foot contact.

## Data Availability

The data presented in this study are available upon request from the corresponding author due to privacy restrictions.

## Abbreviations

The following abbreviations are used in this manuscript:

ACL	Anterior Cruciate Ligament
KAA	Knee Abduction Angle
KAM	Knee Abduction Moment
GRF	Ground Reaction Force
ISN	National Sports Institute of Malaysia
ICC	Intraclass Correlation Coefficient
FC	Foot Contact
SD	Standard Deviation
